# Gene Expression Profiling during Murine Tooth Development

**DOI:** 10.3389/fgene.2012.00139

**Published:** 2012-07-31

**Authors:** Maria A. dos Santos Silva Landin, Maziar Shabestari, Eshrat Babaie, Janne E. Reseland, Harald Osmundsen

**Affiliations:** ^1^Department of Oral Biology, University of OsloOslo, Norway; ^2^Department of Biomaterials, Institute for Clinical Dentistry, University of OsloOslo, Norway; ^3^The Biotechnology Centre of Oslo, University of OsloOslo, Norway

**Keywords:** tooth development, ameloblastin, amelogenin, enamelin

## Abstract

The aim of this study was to describe the expression of genes, including ameloblastin (*Ambn*), amelogenin X chromosome (*Amelx*), and enamelin (*Enam*) during early (pre-secretory) tooth development. The expression of these genes has predominantly been studied at post-secretory stages. Deoxyoligonucleotide microarrays were used to study gene expression during development of the murine first molar tooth germ at 24 h intervals, starting at the 11th embryonic day (E11.5), and up to the 7th day after birth (P7). The profile search function of Spotfire software was used to select genes with similar expression profile as the enamel genes (*Ambn*, *Amelx*, and *Enam*). Microarray results where validated using real-time reverse transcription-polymerase chain reaction (real-time RT-PCR), and translated proteins identified by Western-blotting. *In situ* localization of the *Ambn*, *Amelx*, and *Enam* mRNAs were monitored from E12.5 to E17.5 using deoxyoligonucleotide probes. Bioinformatics analysis was used to associate biological functions with differentially expressed (DE; *p* ≤ 0.05) genes. Microarray results showed a total of 4362 genes including *Ambn*, *Amelx*, and *Enam* to be significant DE throughout the time-course. The expression of the three enamel genes was low at pre-natal stages (E11.5–P0) increasing after birth (P1–P7). Profile search lead to isolation of 87 genes with significantly similar expression to the three enamel proteins. These mRNAs were expressed in dental epithelium and epithelium derived cells. Although expression of *Ambn*, *Amelx*, and *Enam* were lower during early tooth development compared to secretory stages enamel proteins were detectable by Western-blotting. Bioinformatic analysis associated the 87 genes with multiple biological functions. Around 35 genes were associated with 15 transcription factors.

## Introduction

Interactions between oral epithelium and neural crest derived mesenchyme are considered essential for tooth development. Cells of the epithelium expand and proliferate, invaginating into the condensing mesenchyme, and subsequently forms the tooth germ (Thesleff, 1995; Chai et al., 2000; Sharpe, 2001), this process been modulated by several growth factors (Thesleff and Mikkola, 2002; Zhang et al., 2005). As the invaginating epithelium expands it is surrounded by condensing mesenchyme transforming into a bud and cap, subsequently developing into the bell stage (Tucker and Sharpe, 1999; Fleischmannova et al., 2008). Mesenchymal cells facing the basement membrane differentiate into dentin producing odontoblasts, while the adjacent layer of epithelial cells differentiates into ameloblasts which secrete the organic enamel matrix (Thesleff and Hurmerinta, 1981).

Ameloblastin (encoded by *Ambn*) is expressed in the mineralizing matrix of bones and teeth. This matrix protein inhibits ameloblast proliferation and is essential for ameloblast adhesion affecting thickness of the enamel layer (Zhang et al., 2011).

Amelogenins (encoded by *Amel*) are expressed in epithelium derived cells, bone marrow, and mesenchymal stem cells (MSCs). Amelogenins are the main component in the developing enamel matrix and essential for normal enamel thickness and structure (Feng et al., 2012).

Enamelin (encoded by *Enam*) is a minor constituent of the extracellular matrix but plays a critical role in normal enamel formation. Enamelin is required for the deposition of tooth enamel, but it is also necessary to maintain the ameloblast phenotype, as is the case for ameloblastin (Hu et al., 2011).

The expression of about 300 genes has been mapped^1^ and has contributed to our understanding of tooth development. The expression of a substantially higher number of genes is likely to be involved. The use of microarrays facilitates the global mapping of genes at various stages of tooth development.

Recent results obtained from gene expression profiling, between two developmental stages (E15.5 and P2) of murine tooth germs using microarrays indicated that *Amelx*, *Ambn*, and *Enam* exhibited low levels of expression at the studied pre-natal stage (E15.5; Osmundsen et al., 2007). We investigated if these three genes were expressed prior to tooth mineralization and if other genes followed the same expression pattern; low levels of expression at pre-natal stages followed by increase at post-natal stages, during murine tooth development. The study of global gene expression during both pre- and post-natal stages (16 time points) of murine molar tooth development using microarrays should provide data capable of capturing dynamic gene expression profiles during tooth development, combined with bioinformatics might help our understanding of cellular processes underlying development of the murine tooth germ.

## Materials and Methods

### Experimental animals

The day of the vaginal plug was set to embryonic day E0.5 in pregnant female CD-1 mice (*n* = 3–5). The embryo/fetus developmental stage was assessed using the Theiler criteria (Kaufman, 1992).

The animal house had 12 h light/dark cycle and was thermostated at 21°C with relative humidity at 55 ± 5%. Water and fodder was provided *ad lib*. The animals were housed according to the regulations of the Norwegian Gene Technology Act of 1994.

Female mice pregnant at various stages were sacrificed by cervical dislocation and embryos quickly removed from the amnion sac and decapitated.

### Tissue preparation

The first mandibular molar tooth germs was dissected for microarray and real-time RT-PCR analysis from CD-1 embryos at various developmental stages starting at the 11th embryonic day (E11.5) and ending at the 7th day after birth (P7).

For *in situ* hybridization (ISH) embryos heads were dissected and fixed in 4% (v/v) cold, neutral buffered formalin (NBF; AppliChem, Darmstadt, Germany). Heads dissected at P2 up to P7 were decalcified in 12.5% (w/v) ethylenediaminetetraacetic acid (EDTA), 2.5% (v/v) formalin. Paraffin embedding was carried out essentially as described by Hougaard et al. (1997). Serial sections 8 μm thick were used.

Batches of three to nine molar tooth germs were dissected for Western blot analysis at each of the various developmental time points used in this investigation. The tooth germs were lysed using CelLytic MT (Sigma, St Louis, MO, USA) and Halt Protease Inhibitor Cocktail (Pierce Biotechnology, Rockford, IL, USA). Protein concentrations were assayed using Bio-Rad Protein Assay kit (Bio-Rad Laboratories, Hercules, CA, USA).

### Microarray analysis of mRNAs isolated from tooth germs

Total RNA was extracted from tooth germs as described previously by Osmundsen et al. Murine deoxyoligonucleotide (30 k)-microarrays printed with Operon murine v.3 oligo-set (Qiagen GmbH, Hilden, Germany) were purchased from the NTNU Microarray Core Facility (Norwegian University of Science and Technology, Trondheim, Norway). Spikes from *A*. *thaliana* (purchased from Stratagene, La Jolla, CA, USA) were used to normalize the fluorescence within each microarray and to monitor the quality of the hybridization. Complementary DNA synthesis, labeling, and hybridization were carried out as described previously (Osmundsen et al., 2007).

After hybridization and scanning the resulting expression data (48 arrays) was assembled into a single data file. Cy3 and Cy5 channels of each slide were treated as single channel data as if derived from single color arrays to facilitate statistical and bioinformatic analysis. The genes exhibiting a net fluorescence fewer than 200 were excluded. LOESS normalized fluorescence intensities (median values, with background subtracted) from each of the two channels were converted to log2-scale, and the log2-values were subjected to *z*-score normalization (Cheadle et al., 2003).

Statistical analysis of microarray data was carried out using Spotfire v. 9™ Decision Site for Microarray Analysis (Spotfire, MA, USA) from sets of three arrays at each time point.

The ANOVA facility of the Spotfire program was used to select genes which exhibited statistically significant differences in levels of expression (*p* < 0.05) between various developmental stages. False discovery rate (FDR; 0.05; Benjamini and Hochberg, 1995; Reiner et al., 2003) was used to correct selection of genes for false positives.

Experimental design and resulting microarray files have been deposited in the MIAME database with reference E-MEXP-3581.

### Isolation of genes using profile search

Profile search function of Spotfire software v.9 Decision Site for Microarray analysis software (TIBCO Spotfire, Somerville, MA, USA) was used to select differentially expressed (DE) genes with a similar expression pattern to that of pre-selected genes *Ambn*, *Amelx*, and *Enam*. The mean time-course for *Ambn*, *Amelx*, and *Enam* genes (normalized data) throughout the studied time-course was used as search criteria (master profile). The resulting expression profile was subjected to hierarchical clustering and the result presented as heat map. Unknown genes without an Entrez ID were omitted from this analysis.

### Real-time RT-PCR

Triplicates of tooth germs for each time point were used in cDNA synthesis (Fermentas, St. Leon Route, Germany). The subsequent real-time PCR was carried out in a Stratagene MX3005P (Stratagene, La Jolla, CA, USA), using SYBR Green-based assay Ampliqon III (Ampliqon, Rødovre, Denmark). Real-time RT-PCR data was analyzed using the 2^−ΔΔCt^ method 2[−Delta Delta C(T); Pfaffl, 2001]. Where ΔΔCt = (Ct_target_ − Ct_Rpl27_)_Time point *x*_ − (Ct_target_ − Ct_Rpl27_)_E11.5_. Where time *x* = E11.5 up to P7. All time points where compared to E11.5 and normalized against ribosomal protein L27 (Rpl27; Pfaffl, 2001). The primer sequences are listed in Table 1.

**Table 1:** **Sequences of primers used for real-time RT-PCR assays**.

Gene	Sequence of left primer	Sequence of right primer
Amelx	5′-CTC TGC CTC CAC TGT TCT CC-3′	5′-ACT TCT TCC CGC TTG GTC TT-3′
Enam	5′-GCT TTG GCT CCA ATT CAA AA-3′	5′-AGG ACT TTC AGT GGG TGT-3′
Ambn	5′-CTG TTA CCA AAG GCC CTG AA-3′	5′-GCC ATT TGT GAA AGG AGA GC-3′
Rpl27	5′-GGG AAA GTG GTG GTG GTG CT-3′	5′-CAC CAG GGC ATG GCT GTA AG-3′
Wif1	5′-ACC CTG CCG AAA TGG AGG T-3′	5′-TTG GGT TCG TGG CAG GTT C-3′
Krt17	5′-TGT TGG ATG TGA AGA CAA GG-3′	5′-TGA GTC CTT AAC GGG TGG TC-3′
Clu	5′CAC ATG TCT CCA GGC GAG TA-3′	5′ATC AGT TCT TCC CGA GAG CA-3′
Prnp	5′-TTC AGG TCC CTT TGA TGG AA-3′	5′-CCA AAA CAA AGC CCA ACT A-3′
MMP20	5′-AGG GAC GAA GAG AGC TGT GA-3′	5′-AAC CTT CAA CCC TCA CG-3′
Col1a2	5′-GTC CTA GTC GAT GGC TGC TC-3′	5′-CAA TGT CCA GAG GTG CAA TG-3′
Wint4	5′-ACA GCT GGA GGG CTGA CTA A-3′	5′-GTA TGT CCC TTG GGG AAC CT-3′
Wint6	5′-CTA TCC AGG CCT TGG GAA AT-3′	5′-CCT GCA GGA ACTA GCA AAG G-3′
TGFB1	5′-GTG TGG AGC AAC ATG TGG AA-3′	5′-CGT CAA AAG ACA GCCA CTCA-3′
SerpinB5	5-′AAC CAG TCC AAC TCG ACC AC-3′	5′-TGC TCA TAA AGT CGG TGC TG-3′

### Bioinformatic analysis of differentially expressed genes

Bioinformatic analysis using Ingenuity Pathway Analysis (IPA; Ingenuity Systems Inc., Redwood City, CA, USA) was carried out to identify significant associations (Fisher’s Exact Test, *p* ≤ 0.05) with canonical pathways, signaling pathways, transcription factors, molecular, and cellular functions for the genes DE, isolated using the profile search function of the Spotfire software. Transcription factors with *p*-value of overlap <0.01 where considered to be significantly associated with the DE expressed genes.

### *In situ* hybridization

*In situ* hybridization was used to visualize microarray and real-Time RT-PCR results for the genes of the expression profile and was carried out using a protocol essentially as described by Farquharson et al. (1990). ISH was performed in a Discovery XT hybridization instrument (Ventana Medical Systems, AZ, USA) according to the Ventana recommendations.^2^ The *Ambn* anti-sense probe, 5′-ggtttcagtcctggctggtgaggctgcaaggatggctgctgggtttca-3′, hybridizes to nucleotides 378–425 located within the coding sequence of NM_009664.

The *Amelx* probe, 5′-ggacaggggctgcatggagaacagtggaggcagaggtggctgtggtgc-3′, hybridizes to nucleotides 544–591 within the coding sequence of NM_009666. The *Enam* probe, 5′-tgtggctgtggctcttgtggattggtctggttgggtttggcggtctcc-3′ hybridizes to nucleotides 672–719 located within the coding sequence of NM_017468. The probe concentration was 0. 45 μg μl ^−1^. The hybridization temperature was set to 57°C. Antibodies (diluted 1:2000) purchased from Jacksons Immuno (Jackson Immuno Research Europe, Suffolk, UK) and BlueMap kit (NBT/BCIP; Ventana Medical Systems) was used for detection and visualization. No counter-staining was used following ISH.

The quality of mRNA in the tissue was assessed using digoxigenin (DIG)-labeled poly dT deoxyoligonucleotide probes. The nature of the hybridization signals was ascertained by treating separate tissue sections prior to pre-hybridization with either RNAse (RNAseA, Qiagen, Hilden, Germany) or DNAseI (DNA-free™ Ambion, CA, USA). RNAse was used at a final concentration of 20 μg RNAseA ml^−1^, the tissue sections being incubated for 30–120 min at 37°C. The DNAse treatment was performed for 15–30 min at 90°C using a final concentration of 5 μg DNAse I μl^−1^. ISH micrographs were obtained using a Nicon Elipse E400 instrument (Nikon Corporation, Tokyo, Japan).

### Western-blotting

Aliquots containing 100 μg of total protein were separated by sodium dodecylsulfate-polyacrylamide gel electrophoresis (SDS-PAGE) using ReadyGel (4–20%) with Tris-HCl buffer (Bio-Rad Laboratories). Proteins were blotted onto 0.45 μm Trans-blot nitrocellulose membranes (Bio-Rad Laboratories) and incubated with antibodies (1:500) against Amelogenin X (SC-32892), Ameloblastin (N-18; SC-33100), or Enamelin (C-18; SC-33107; Santa Cruz Biotechnology, CA, USA), using goat anti-rabbit as secondary antibody (1:5000; Vector Laboratories, CA, USA). Enzymatic activity was detected by Enhanced Chemifluorescence (ECF) substrate (GE Healthcare, Bucks, UK) using the Storm 860 imaging scanner (GE Healthcare). The specificity of the antibodies was tested incubating with only the secondary antibody.

## Results

### Microarray analysis of mRNAs isolated from tooth germs

**Figure 1:**
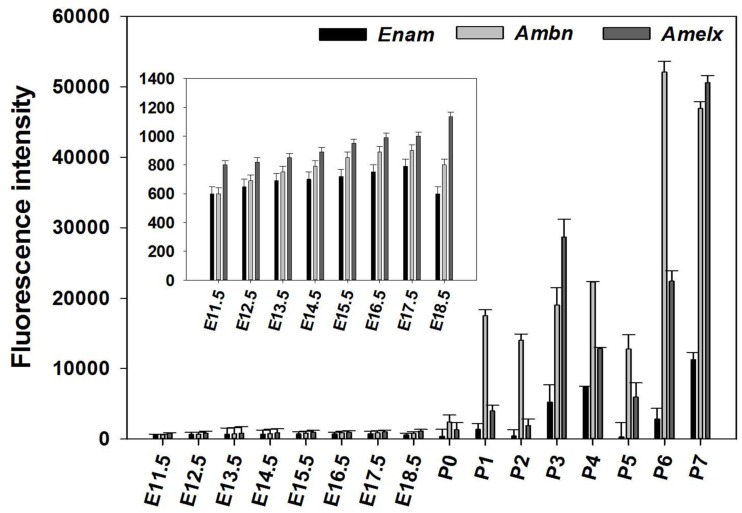
**Expression pattern of *Amelx*, *Ambn*, and *Enam* during development of the molar tooth germ**. Expression of *Ambn*, *Amelx*, and *Enam* mRNA monitored using deoxyoligonucleotide microarrays at the various stages of murine tooth development. The plotted normalized net fluorescence intensities (raw fluorescence intensity minus background) are means derived from biological triplicates at each time point with SD indicated. Expression pattern showed low fluorescence intensities at the pre-natal stages (E11.5–P0), with increasing signal throughout the post-natal stages (P1–P7).

*Ambn*, *Amelx*, and *Enam* had similar expression pattern throughout the developmental stages (Figure 1), showing low pre-natal levels of expression (net fluorescence intensities of about 800). At P1–P5 about 33-fold increase in fluorescence intensity was observed, further increasing to 100-fold at P6–P7, compared to the pre-natal stages (Figure 1).

**Figure 2:**
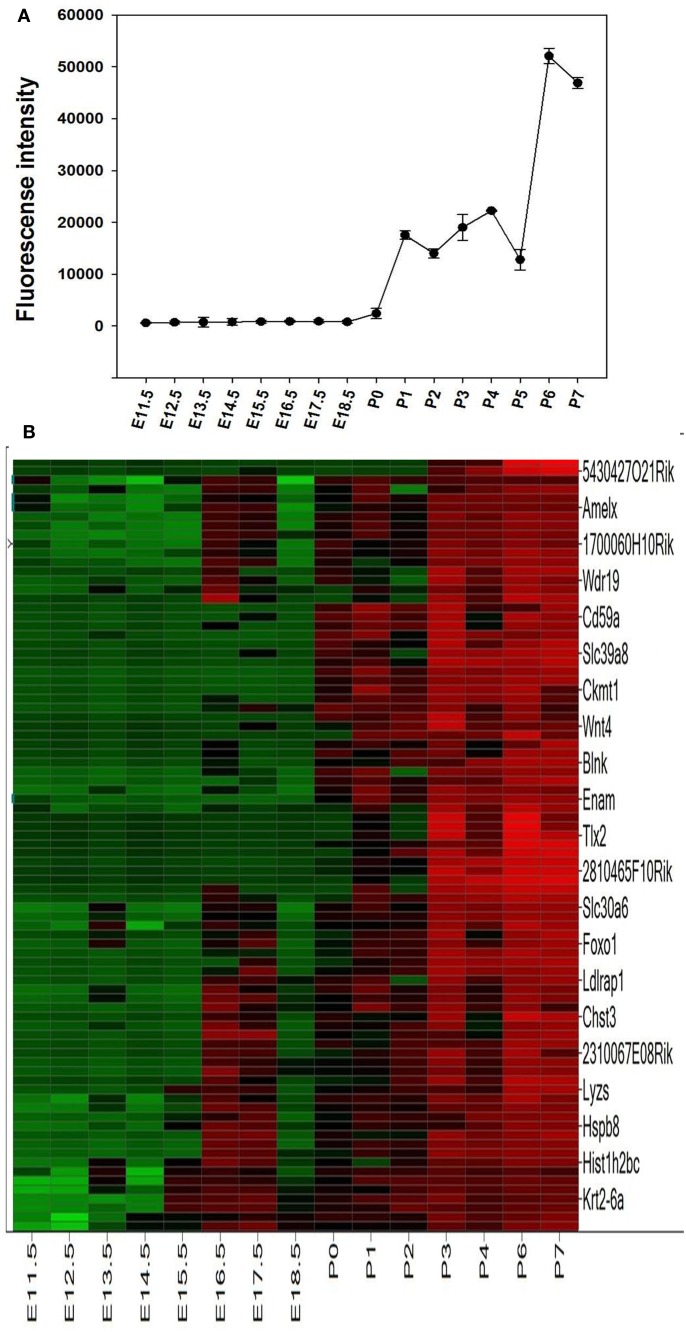
**Profile search results**. The mean net normalized fluorescence intensities for *Ambn*, *Amelx*, and *Enam* genes **(A)** at each time point was used as search criteria (master profile) for profile search. The resulting 87 genes were hierarchically clustered using the Euclidean distance and the result is presented as heat map **(B)**. Columns represent different developmental stages and rows represent each gene. Each cell in the matrix represents the expression level of a gene at a certain time point/developmental stage. Green and red in cells reflect low and high expression levels, respectively.

The microarray results showed a total of 4362 genes to be differentially (*p* ≤ 0.05) expressed at every time point studied (E11.5–P7), 1169 of which being without an Entrez ID and were consequently not used during further analysis. The remaining 2441 genes were used in a profile search using the mean time-course of expression for *Ambn*, *Amelx*, and *Enam* (Figure 2A). From the 2441 genes screened with the profile search function in Spotfire, 87 genes (Figure 2B) exhibited at time-course of expression similar that of *Amelx*, *Ambn*, and *Enam*.

### Real-time RT-PCR

**Figure 3:**
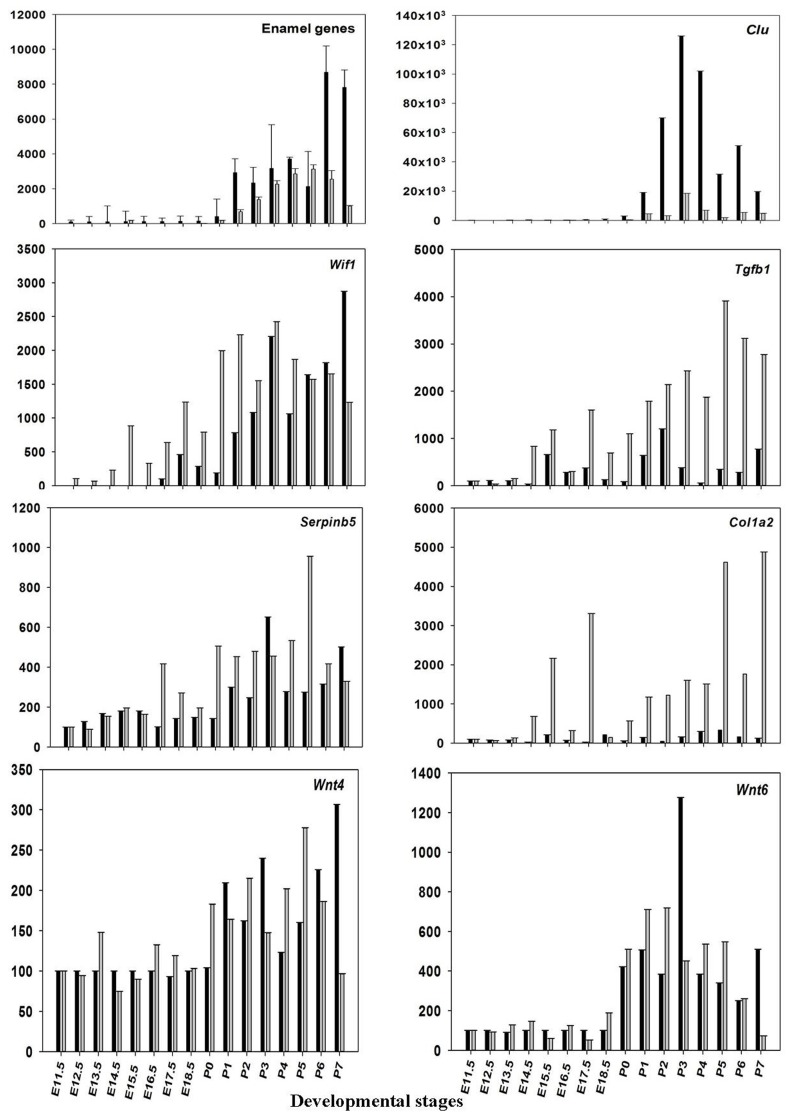
**Validation of microarray data**. The mean ratios (expression at E11.5 up to P7/expression at E11.5) for some of the differentially expressed (DE) genes (*Amelx*, *Ambn*, *Enam*, *Wif1*, *Clu*, *Prnp*, *Mmp20*, *Col1a1*, *Tgfb1*, *Wint6*, and *Serpinb5*) from the microarray data (black) and real-time RT-PCR (gray). Plotted microarray and real-time RT-PCR data are means derived from at least three biological triplicates at each time point and presented with SD.

The microarray results of mRNA expression for the enamel genes *Ambn*, *Amelx*, *Enam*, and some random genes from the profile search clusterin (*Clu*), Wnt inhibitory factor 1 (*Wif1*), keratin 17 (*Krt17*), prion protein (*Prnp*), matrix metallopeptidase 20 (*Mmp20*), Collagen type I, alpha 2 (*Col1a2*), transforming growth factor, beta 1(*Tgfb1*), wingless-related MMTV integration site 4 (*Wint4*), wingless-related MMTV integration site 6 (*Wint6*), and serine (or cysteine) peptidase inhibitor, clade B, member 5(*Serpinb5*), were verified using real-time RT-PCR. The results show good agreement with the results obtained from microarray (Figure 3 and Table 2), confirming that pre-natal levels of mRNAs of *Amelx*, *Ambn*, *Enam*, *Wif1*, *Krt17*, *Clu*, *Prnp*, *Mmp20*, *Col1a1*, *Tgfb1*, *Wint4*, *Wint6*, and *Serpinb5* are markedly lower compared to post-natal levels (Table 2). The microarray and real-time RT-PCR results showed good agreement.

**Table 2:** **Expression of genes measured with real-time RT-PCR**.

	*Amelx*	*Ambn*	*Enam*	*Wif1*	*Krt17*	*Clu*	*Prnp*	*MMP20*	*Col1a2*	*Wint4*	*Wint6*	*TGFB1*	*SerpinB5*
E11.5	1.0 ± 0.10	1.0 ± 0.30	1.0 ± 0.70	1.0 ± 0.05	1.0 ± 0.05	1.0 ± 0.43	1.0 ± 0.020	1.0 ± 0.038	1.0 ± 0.011	1.0 ± 0.076	1 ± 0.012	1.0 ± 0.072	1.00 ± 0.033
E12.5	1.2 ± 0.20	1.6 ± 0.50	1.3 ± 0.60	66.15 ± 0.08	0.41 ± 0.23	1.4 ± 0.20	1.3 ± 0.22	0.423 ± 0.033	0.69 ± 0.016	0.945 ± 0.077	0.917 ± 0.545	0.372 ± 0.016	0.881 ± 0.451
E13.5	1.1 ± 0.21	2.1 ± 0.40	1.9 ± 1.10	2.29 ± 0.21	0.49 ± 0.13	1.9 ± 0.16	2.6 ± 0.024	0.499 ± 0.225	1.342 ± 0.064	1.479 ± 0.116	1.281 ± 0.115	1.525 ± 0.04	1.532 ± 0.08
E14.5	179.1 ± 15	21.1 ± 3.50	12.4 ± 7.50	8.85 ± 1.40	1.03 ± 0.18	3.5 ± 9.43	5.3 ± 0.040	0.71 ± 0.302	6.792 ± 0.033	0.748 ± 0.095	1.457 ± 0.162	8.342 ± 0.03	1.957 ± 0.032
E15.5	0.4 ± 0.10	2.3 ± 0.50	0.4 ± 0.20	3.32 ± 0.27	0.82 ± 0.35	1.7 ± 13.91	2 ± 0.010	1.628 ± 0.465	21.672 ± 0.569	0.898 ± 0.068	0.593 ± 0.095	11.815 ± 0.08	1.639 ± 0.432
E16.5	1.3 ± 0.12	2 ± 0.40	1.5 ± 1.30	6.36 ± 0.73	1.07 ± 0.15	1.26 ± 11.89	3 ± 0.050	1.87 ± 0.232	3.22 ± 0.015	1.323 ± 0.107	1.247 ± 0.308	3.022 ± 0.025	4.161 ± 0.016
E17.5	0.7 ± 0.005	1.7 ± 0.60	1.1 ± 0.50	12.36 ± 1.19	3.72 ± 0.41	0.65 ± 0.72	5.8 ± 0.051	0.767 ± 0.26	33.06 ± 0.017	1.192 ± 0.095	0.518 ± 0.03	15.993 ± 0.17	2.704 ± 0.032
E18.5	7.1 ± 1.10	2.9 ± 5.00	1.2 ± 0.60	7.9 ± 0.47	2.37 ± 0.33	0.61 ± 0.38	8.4 ± 1.10	0.985 ± 0.032	1.423 ± 0.016	1.031 ± 0.086	1.887 ± 0.38	6.928 ± 0.34	1.957 ± 0.042
P0	175.4 ± 16	26.3 ± 11	97.2 ± 45	1994.23 ± 3.89	376.14 ± 0.28	506.53 ± 8.20	11 ± 0.073	5.016 ± 1.166	5.693 ± 0.013	1.828 ± 0.149	5.104 ± 0.15	10.986 ± 0.32	5.053 ± 0.332
P1	679.8 ± 120	55 ± 0.75	278.1 ± 124.5	2229.14 ± 1.33	515.86 ± 0.32	4584.88 ± 20.69	11.2 ± 0.05	15.78 ± 0.619	11.763 ± 0.161	1.641 ± 0.132	7.1 ± 0.027	17.884 ± 0.44	4.53 ± 0.065
P2	1373 ± 150	384.9 ± 76	750.7 ± 356	1550.02 ± 1.44	503.96 ± 0.06	3305.16 ± 6.41	8.7 ± 0.073	35.18 ± 9.052	12.203 ± 0.468	2.148 ± 0.017	7.186 ± 0.17	21.37 ± 0.47	4.789 ± 0.016
P3	2258 ± 210	636.3 ± 180	1051 ± 476.5	2420.65 ± 2.18	1171.15 ± 0.21	18521.99 ± 5.29	14.1 ± 1.40	84.84 ± 18.301	16.018 ± .078	1.474 ± 0.012	4.51 ± 0.016	24.31 ± 0.33	4.552 ± 0.157
P4	2855 ± 300	954.1 ± 150	1431 ± 660.5	1866.26 ± 1.21	51.47 ± 0.22	6974.91 ± 19.91	13.4 ± 1.30	117.241 ± 17.447	15.117 ± 0.16	2.019 ± 0.17	5.357 ± 0.27	18.719 ± 0.18	5.332 ± 0.33
P5	3125 ± 245	753.8 ± 110	958.5 ± 477.5	1571.36 ± 0.56	102.21 ± 0.09	1995.45 ± 5.81	15 ± 1.40	84.84 ± 21.832	46.165 ± 0.78	2.775 ± 0.227	5.474 ± 0.04	39.041 ± 0.37	9.551 ± 0.16
P6	2541 ± 500	599.7 ± 76	488.8 ± 153	1651.76 ± 1.2	120.99 ± 0.06	5461.11 ± 6.28	22 ± 2.30	115.36 ± 24.376	17.635 ± 0.86	1.863 ± 0.016	2.609 ± 0.075	31.166 ± 0.16	4.167 ± 0.37
P7	1004 ± 45	346 ± 50	249.8 ± 79	1232.94 ± 0.47	600.23 ± 0.35	4814.55 ± 10.25	7.4 ± 0.90	24.477 ± 2.145	48.807 ± 0.163	0.966 ± 0.079	0.725 ± 0.099	27.741 ± 0.31	3.286 ± 0.17

*The tabulated values represent mean fold changes in expression (relative to levels of expression at E11.5) derived from assays on three separate batches of cDNA (RNA having been isolated from separate batches of tooth germs) with SD indicated. All assays were carried out using biological triplicates*.

### Bioinformatic analysis of differentially expressed genes

These 87 genes (Figure 2B) submitted to bioinformatic analysis using IPA were found to be significantly associated with the Ingenuity categories called “gene expression,” “tissue development” (Table 2), “embryonic, organ, and cellular development,” “cell-to-cell signaling and interaction” (*Tgfb1*, *Wnt4*, *Agrn*, *Atp1b1*, *Serpinb5*, *Ambn*, *Clu*, Tgfb1), “cellular growth and proliferation,” “hair and skin development and function” (*Cldn*1, *Cst6*, *Sfn*, *Tgfb1*, *Wnt4*), “dental disease” (*Amelx*, *Enam*, *Klk4*, *Mmp20*; Table 3). Transcription factor analysis suggested that 15 transcription regulators.

**Table 3:** **The 87 genes isolated using profile search were investigated using IPA to determine significant associations (*p* = 0.01) with “Category” or with “Functions” as judged by Fisher’s exact test**.

Category	Functions	Molecules	*p*-Value	# Molecules
Gene expression	Transcription of DNA	Aebp1, Agrn, Bglap2, Blnk, Foxo1, Pdlim1, Pml, Sp6, Tgfb1, Tlx2, Wnt4, Wnt6	1.36E−02	12
Tissue development	Development of skeletal system	Col11a2, Ltbp3, Tgfb1	9.88E−03	3
	Developmental process of enamel	Klk4, Mmp20	9.35E−05	2
	Aggregation of cells	Amelx, Atp1b1, Clu, Tgfb1	9.33E−03	4
	Adhesion of neuronal cells	Agrn, Atp1b1	9.04E−03	2
	Tissue development	Aebp1, Agrn, Amelx, Aplp1, Bglap2, Calb1, Chst3, Cldn1, Col11a2, Cst6, Cyp26a1, Foxo1, Kcnj8, Ltbp3, Mmp20, Pml, Prnp, Serpinb5, Sfn, Tgfb1, Tlx2, Wnt4, Wnt6	6.55E−07	23
	Organization of tissue	Ambn, Aplp1, Serpinb5, Tgfb1	3.96E−03	4
	Adhesion of connective tissue cells	Ambn, Clu, Tgfb1	3.85E−03	3
	Development of embryonic tissue	Cyp26a1, Serpinb5, Tgfb1, Tlx2, Wnt4, Wnt6	3.44E−03	6
	Formation of connective tissue	Col11a2, Cyp26a1	3.00E−03	2
	clustering of cells	Clu, Tgfb1	2.30E−03	2
	Deposition of extracellular matrix	Serpinb5, Tgfb1	2.05E−03	2
	Development of connective tissue	Calb1, Chst3, Col11a2, Cyp26a1, Foxo1, Ltbp3, Pml, Tgfb1	1.77E−03	8
	Formation of tissue	Chst3, Col11a2, Cyp26a1, Foxo1, Ltbp3, Tgfb1, Tlx2	1.48E−03	7
	Morphogenesis of tissue	Ltbp3, Serpinb5, Tgfb1, Wnt4, Wnt6	1.36E−03	5
Embryonic development	Organogenesis	Aebp1, Amelx, Aplp1, Bglap2, Cldn1, Cst6, Cyp26a1, Foxo1, Kcnj8, Mmp20, Serpinb5, Sfn, Tgfb1, Wnt4, Wnt6	1.25E−04	15
Organ development	Aging	Clu, Pml, Prnp	1.84E−04	3
Cellular development	Differentiation of bone marrow cells	Pml, Tgfb1, Wnt4	9.64E−03	3
	Maturation of cells	Blnk, Clu, Pml, Prnp, Tgfb1	8.78E−03	5
	Differentiation of cells	Agrn, Ambn, Blnk, Clu, Col11a2, Cyp26a1, Ltbp3, Ndrg1, Plac8, Pml, Prnp, Sfn, Sp6	5.40E−04	16
	Differentiation of mesenchymal cells	Foxo1, Tgfb1	3.75E−03	2
	Differentiation of mesenchymal stem cells	Tgfb1, Wnt4	3.13E−03	2
Cell-to-cell signaling and interaction	Adhesion of neuronal cells	Agrn, Atp1b1	9.04E−03	2
	Adhesion of endodermal cells	Serpinb5	3.99E−03	1
	Adhesion of connective tissue cells	Ambn, Clu, Tgfb1	3.85E−03	3
Cell signaling	Retinoic acid receptor signaling	Cyp26a1, Pml	5.54E−04	2
Cellular assembly and organization	Opening of pore	Ckb, Ckmt1a/Ckmt1b	4.69E−05	2
	Deposition of amyloid fibrils	Clu, Tgfb1	1.59E−03	2
Cellular growth and proliferation	Proliferation of smooth muscle cells	Clu, Serpinb5, Tgfb1	7.42E−03	3
	Growth of fibroblast cell lines	Hspb8, Ldlrap1, Pml, Tgfb1	7.33E−03	4
	Proliferation of connective tissue cells	Aebp1, Ambn, Foxo1, Pml, Tgfb1	6.73E−03	5
	Proliferation of muscle cells	Clu, Prnp, Serpinb5, Tgfb1	2.66E−03	4
	Proliferation of epithelial cells	Ambn, Serpinb5, Sfn, Sp6, Tgfb1	2.34E−03	5
Cell death	Cell cycle progression	Blnk, Clu, Foxo1, Pml, Serpinb5, Sfn, Tgfb1	8.65E−03	7
Hair and skin development and function	Skin development	Cldn1, Cst6, Sfn, Tgfb1, Wnt4	6.07E−04	5
Tissue morphology	Repair of tissue	Ambn, Tgfb1	3.44E−03	2
	Differentiation of epithelial cell lines	Sp6, Tgfb1	2.05E−03	2
Lipid metabolism	Metabolism of lipid	Calb1, Clu, Cyp26a1, Foxo1, Gm2a, Lrp10, Ppap2a, Prnp, Tgfb1	9.80E−04	9
	Synthesis of lipid	Aebp1, Clu, Cyp26a1, Foxo1, Mc4r, Tgfb1, Calb1, Foxo1, Ppap2a, Prnp, Tgfb1, Wnt4	3.87E−03	7
Dental disease		Amelx, Enam, Klk4, Mmmp20	3.65E−03	
Developmental disorder		Amelx, Aplp1, Clu, Col1a2, Cyp26a1, Hspb8, Mc4r, Pml, Tgfb1	1.91E−03	9

**Figure 4:**
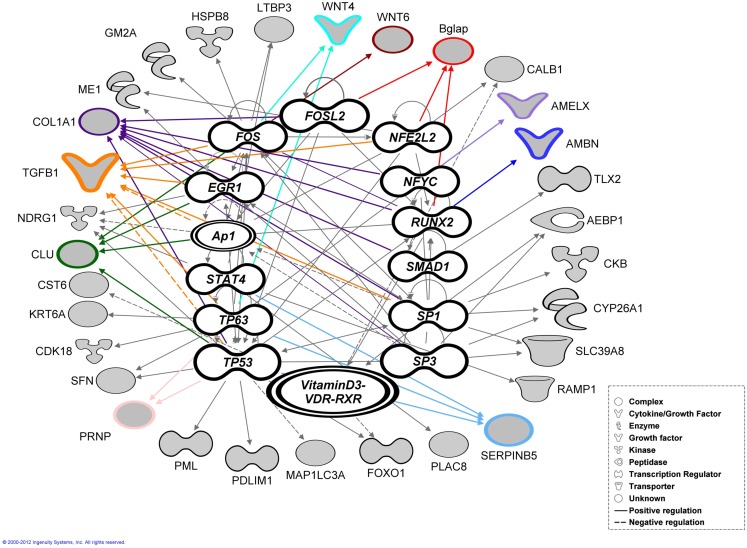
**Bioinformatic analysis**. Transcription factor analysis performed using ingenuity (IPA) for the 87 genes exhibited a *p*-value of overlap <0.01 associating significantly 35 genes with 15 transcription factors. The genes verified by real-time RT-PCR highlighted with colored outlines and lines together with most of the associated genes (shaded gray).

*The genes shown in the table exhibited similar levels of expression to the preselected genes of the master profile*.

(Egr1, Fos, Folsl2, Hif1a, Nfel2, Nfya Runx2, Smad1, Ap1 Sp1,Sp3, Stat4, Tp53, Tp63, VitaminD3-VDR-RXR) exhibited a *p*-value of overlap <0.01, proposing significant regulatory associations to several of the 87 cluster genes (Figure 4).

### *In situ* hybridization

**Figure 5:**
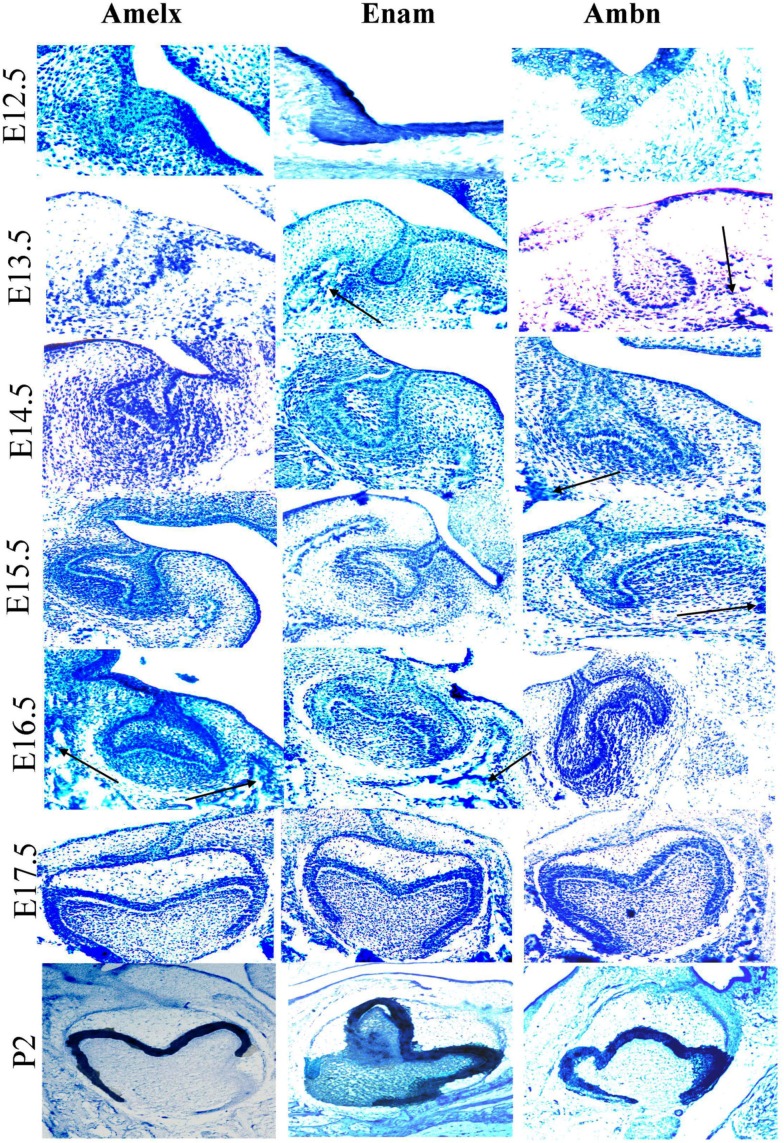
**Localization of expression of *Ambn*, *Amelx*, and *Enam* mRNAs during early odontogenesis as detected by *in situ* hybridization**. Detection of *Amelx*, *Enam*, and *Ambn* mRNA was carried out in dissected sections of tooth germs at various stages of development (E12.5–P2). The black arrows indicate mRNA expressed at future facial bone features. The magnifications were ×200 for E12.5–E13.5, ×100 for E14.5 and E15.5–P2.

During early odontogenesis *Amelx*, *Ambn*, and *Enam* mRNAs showed similar patterns of localization and were mainly observed in cells derived from dental epithelium (Figure 5). These three mRNAs were, however, also detected in the mesenchyme and mesenchyme-derived tissues. Expression of *Enam* and *Ambn* mRNA were also observed in cells known to form facial bone structures (Figure 5 arrows). In general the signal from the *Amelx* probe appeared stronger than signals from the *Ambn* or *Enam* probes. During the cap stage the area of the enamel knot exhibited a hybridization signal with *Amelx* only (Figure 5, E14.5). Treatment with DNase did not alter the hybridization signal. Hybridization with the sense probes consistently showed absence of positive signals (results not shown). Sections treated with RNAse showed no hybridization signals, suggesting the signal to originate from hybridization with RNA in the tissue (results not shown).

### Western-blotting

**Figure 6:**
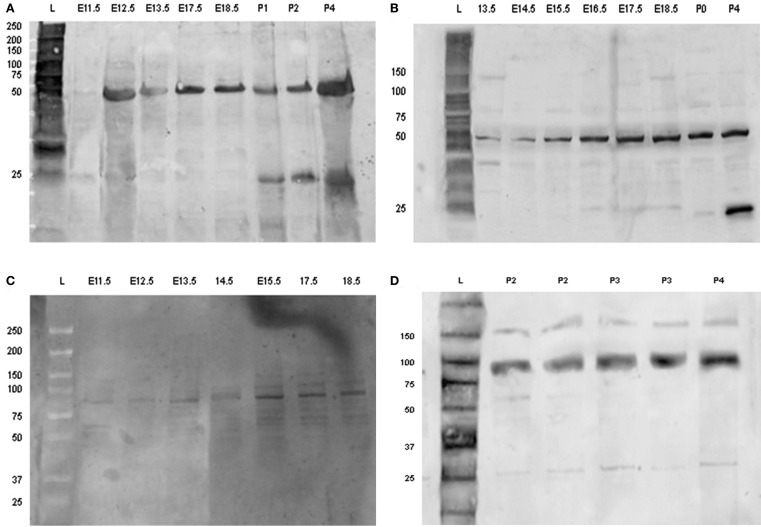
**Detection of amelogenin and enamel in using Western-blotting**. Western blot of amelogenin **(A,B)** and enamelin **(C,D)** from tooth germs dissected from E11.5 up to P5. Each sample loaded on to the gels was derived from separate tooth germs. Abbreviations: L, molecular weight standard ladder; E11.5–E18.5, embryonic days 11.5–18.5; P0, birth; P1–P5, post-natal day 1–5.

Amelogenin was identified as a band of approximately 50 kDa detected at all developmental stages investigated, whereas an additional band of amelogenin of molecular weight of about 25 kDa was detected at post-natal stages, being the predominant protein band at P2–4 (Figures 6A,B).

Enamelin was detected at pre-secretory stages as a band with molecular mass of about 89 kDa (Figure 6C). The intensity of this band increased at the later stages of tooth development, together with an additional band of molecular mass of about 60–64 kDa (Figure 6D).

The ameloblastin antibody identified a single band of molecular weight of about 48 kDa at pre-secretory stages. This band was relatively weak at all time points (results not shown).

## Discussion

To the authors knowledge there are no systematic analysis of gene expression throughout the entire time-course of murine tooth development. Several studies focus on gene expression profiles of selected genes, and or between two time points during murine tooth development (Osmundsen et al., 2007; Kim et al., 2012). Here we present global gene expression across a range of time points (E11.5–P7) during tooth development. The expression of several genes, including *Amelx*, *Ambn*, *Enam*, *Wif1*, *Clu*, *Prnp*, *Mmp20*, *Col1a2*, *Tgfb1*, *Wint6*, and *Serpinb5* at pre-secretory stages has not been described earlier. The resulting data indicate that some of the genes mentioned above are expressed in detectable levels at stages prior to the secretory and late maturation stages of murine tooth development.

During early tooth development invagination of the dental epithelium and condensation of the mesenchyme requires fine control and modulation of both short and long-range cellular communication. Members of both fibroblast growth factor (*FGF*) and wingless-related MMTV integration site (*Wnt*) families are expressed in the dental epithelium and have been proposed to regulate gene expression in the underlying mesenchyme during early odontogenesis (Kettunen et al., 2005), e.g., *Wif1* is known to bind through the WIF domain to several *Wnt*s, e.g., *Wint4* controlling and modulating gradients of secreted morphogens which in turn regulate cellular communication during early tooth formation. At post-natal stages these genes play an active role in cell differentiation, e.g., *Wint6* facilitate mineralizing of the murine tooth (Wang et al., 2010). Bioinformatic analysis suggests that some molecules; *Amelx*, *Ambn*, *Clu*, *Tgfb1*, *Serpinb5* have multiple functions, playing different roles during early murine tooth development and mineralization. Most of the genes resulting from *Ambn*, *Amelx*, and *Enam* profile search play an important role in cell proliferation, migration, adhesion during early tooth development (E11.5–E16.5) while during the differentiation and mineralization stages (E17.5 up to P7) these genes contribute to attachment, organization, polarity of either ameloblasts or odontoblasts (Karavanova et al., 1992; Aberg et al., 2004; Nakamura et al., 2008). This multifunctionality may be due to the fact that expression of many of the 87 genes are induced or regulated by different transcription factors and regulators, e.g., Runx2 (*Ambn*, *Bglap*, *Ibsp*, and *Col1a2*), Fosl2 (*Bglap*, *Col1a2*), and VitaminD3/-VDR-RXR (*Calb1*, *CST6*, *FOXO1*), triggering different cellular responses during tooth development (McMahon et al., 1990; Fukumoto et al., 2004; Rufini et al., 2011; Romano et al., 2012). Runx2 suppresses the expression of *Ambn* and *Amel* in cultured tooth germs (Kobayashi et al., 2006). Fos is associated with a variety of biological processes, e.g., proliferation and differentiation. Fosl2 (Fra-2) knockout mice show an aberrant tooth formation (Smeyne et al., 1993). Dietary deficiencies in Vitamin D3 affect Calbindin (*Calb1*) expression in ameloblasts and odontoblasts in rat incisors leading to hypocalcemia and dentin hypomineralization (Berdal et al., 1995).

The *in situ* results for *Amelx*, *Amb*n, and *Enam* mRNA showed similarities with immunohistochemical localization of Serpinb5, Clusterin, and Prnp (French et al., 1993; Davaadorj et al., 2010; Khan et al., 2010). Expression of enamel proteins in bone cells has previously been reported (Spahr et al., 2006; Tamburstuen et al., 2010; Tamburstuen et al., 2011) and is supported by the presented *in situ* data.

The combination of several techniques like microarrays, real-time RT-PCR, ISH, and immuno-blotting combined with bioinformatics may serve to provide a more comprehensive view of the cellular consequences of changes in gene expression occurring during murine tooth development. The major challenge is at the level of analysis of microarray data, i.e., isolation of DE genes which are functionally related. The profile search function, used in this study, has shown itself as a useful tool when searching for related genes in large sets of microarray gene expression data. This may also be useful to identify novel genes during murine tooth development.

## Conflict of Interest Statement

The authors declare that the research was conducted in the absence of any commercial or financial relationships that could be construed as a potential conflict of interest.

## Footnotes

^1^http://bite-it.helsinki.fi

^2^http://www.ventanadiscovery.com
